# Healthy Animals, Healthy People: Zoonosis Risk from Animal Contact in Pet Shops, a Systematic Review of the Literature

**DOI:** 10.1371/journal.pone.0089309

**Published:** 2014-02-26

**Authors:** Kate D. Halsby, Amanda L. Walsh, Colin Campbell, Kirsty Hewitt, Dilys Morgan

**Affiliations:** 1 Gastrointestinal, Emerging and Zoonotic Infections Department, Public Health England, London, United Kingdom,; 2 Centre for the Epidemiological Study of Sexually Transmitted Infections and AIDS of Catalonia (CEEISCAT) – ICO, Hospital Universitari Germans Trias i Pujol, Badalona, Spain; 3 London/KSS Specialty School of Public Health, London Deanery, London, United Kingdom; The Australian National University, Australia

## Abstract

**Background:**

Around 67 million pets are owned by households in the United Kingdom, and an increasing number of these are exotic animals. Approximately a third of pets are purchased through retail outlets or direct from breeders. A wide range of infections can be associated with companion animals.

**Objectives:**

This study uses a systematic literature review to describe the transmission of zoonotic disease in humans associated with a pet shop or other location selling pets (incidents of rabies tracebacks and zoonoses from pet food were excluded).

**Data sources:**

PubMed and EMBASE.

**Results:**

Fifty seven separate case reports or incidents were described in the 82 papers that were identified by the systematic review. Summary information on each incident is included in this manuscript. The infections include bacterial, viral and fungal diseases and range in severity from mild to life threatening. Infections associated with birds and rodents were the most commonly reported. Over half of the reports describe incidents in the Americas, and three of these were outbreaks involving more than 50 cases. Many of the incidents identified relate to infections in pet shop employees.

**Limitations:**

This review may have been subject to publication bias, where unusual and unexpected zoonotic infections may be over-represented in peer-reviewed publications. It was also restricted to English-language articles so that pathogens that are more common in non-Western countries, or in more exotic animals not common in Europe and the Americas, may have been under-represented.

**Conclusions/implications:**

A wide spectrum of zoonotic infections are acquired from pet shops. Salmonellosis and psittacosis were the most commonly documented diseases, however more unusual infections such as tularemia also appeared in the review. Given their potential to spread zoonotic infection, it is important that pet shops act to minimise the risk as far as possible.

## Introduction

Rising numbers of household pets, in particular exotic species, means that an increasing number of people are exposed to the risk of acquiring zoonotic disease from companion animals. Around 67 million pets are now owned by UK households, with 13 million households in the UK (48%) owning at least one pet in 2012 [1]. Traditional pets such as dogs and cats remain the most popular (23% of UK households own a dog and 19% of UK households own a cat) [1], however there has been an increased ownership of exotic pets in recent years, though accurate figures are difficult to obtain. This increase is due in part to the 2007 modification to The Dangerous Wild Animals Act 1976 [2]. This act lists animals for which licenses are required in the UK in order to keep the animal as a pet, whilst the modification to the act removed some exotic animals from the list.

A wide range of infections can be associated with companion animals, including parasitic, bacterial, fungal and viral diseases [3]–[5]. Of those transmitted by bites and scratches, pasteurellosis, cat-scratch disease, and various aerobic and anaerobic infections are predominant. Other common infections are gastrointestinal (e.g. campylobacter, salmonella), dermatologic (e.g. dermatophytoses, scabies), respiratory (e.g. psittacosis) and multisystemic (e.g. toxoplasmosis, leishmaniasis) [3].

The top five sources for acquiring a pet are: friend/acquaintance, rescue centre, pet shop, recommended breeder, and private advertisement [6]. There are studies in the literature examining animal infections in pet shops and other retail outlets [7]–[10], but little exploration of human infections arising from these facilities. Whilst owning a pet will always result in a small risk of zoonotic illness to the owners and those that the pet comes into contact with, a sick animal in a pet shop can potentially spread the illness to other animals within the shop, and to a large number of geographically distributed owners as newly purchased pets are taken home. Pet shops can therefore act as a nexus point for zoonotic disease.

## Methods

In September 2012, a systematic literature review was performed in order to identify any reports of human infection acquired (or where the report’s authors inferred that it had been acquired) from a pet shop or other location selling pets, or an animal reported to have been acquired from such a premises.

### Search Strategy and Selection Criteria

Data for this review were identified by searches of PubMed and EMBASE, and through the references of papers identified by the review (references at all stages of publication were considered). We used the following Boolean search statement: (“pet shop” OR “pet store” OR “pet” OR “companion animal”) AND (“zoonoses” OR “zoonosis” OR “Human infection” OR “Human case”). Articles in English were selected (although foreign language publications were accepted where an English abstract was available and contained sufficient information to fulfill the inclusion criteria), and no date restrictions were applied to the searches. (The main PubMed database contains manuscripts dating back to 1966, whilst EMBASE covers manuscripts from 1974 onwards.).

The abstracts of the articles were examined and retained if they referred to: i) human cases of zoonotic infection, with ii) a link to a pet or companion animal. The full text was then examined and retained if reference was made to: i) human cases of zoonotic infection, ii) which came from a pet (or a potential pet), and iii) where the animal had a link to a pet shop or other location that sells or distributes companion animals. The following information was extracted from the articles: zoonosis/agent, country (of infection or report if not known), year of infection (or report if not known), type of animal, setting (e.g. pet store, pet distributor), number of human cases associated with pet shop (or other location selling/distributing companion animals), age of human cases, method of transmission (e.g. bite or scratch), and type of contact (e.g. domestic or occupational). The information was extracted by the principal investigator and reviewed by a co-author.

A number of articles considered during the systematic review described rabid animals which had been sold in pet shops, and the extensive contact tracing for postexposure prophylaxis (PEP) which had to be conducted as a result. These were not included in this review since none of the articles documented a human case of rabies that had arisen from such animals. Further articles considered by the systematic review described cases of zoonotic infection associated with pet food and treats, purchased in pet shops. These were also not included in the review since the inclusion criteria required the pet itself to have a link to the pet shop.

## Results

One thousand and eighty seven papers were identified by the initial systematic literature review. Nine hundred and forty five of these were English-language articles, of which 265 were retained based on abstracts, and 66 met the full text inclusion criteria. The original search also identified 142 foreign language papers, of which five had sufficient information in the English abstract to include the paper in the final review. In addition, twelve potential articles were identified through the references of included papers, of which eleven met the inclusion criteria themselves.

A total of 82 papers fulfilled the criteria of the systematic review.

The results of the literature review are presented in Table 1 (where a particular incident was described by more than one paper in the review, only primary paper(s) are included in the table; articles which discussed the incident only by reference to the primary paper(s) were not included). If the country of the incident was not stated, it was assumed to be the authors’ country. If a year of incident was not given, the year of publication of the paper was used as a proxy. The number of infections refers to the human cases linked to pet shops in each article, not the total number of human cases discussed.

**Table 1:** Cases of zoonoses associated with pet shops identified by the systematic literature review.

Zoonosis/agent	Country	Year	Animal	Setting	Human casesassociatedwith pet shops	Age: child(≤16 years)/adult	Transmission	Probable type ofcontact: Occ/dom/visitor*	Comment	Main ref
Bartonellosis	USA	1994	Cats	Animalshelter	1 case	Adult	Multiplescratches	>1 category	Case adopted kittens from animal shelter.Case had high antibody titres to*Bartonella henselae.* The kittens were blood culture positive.	[30]
Blastomycosis	USA	2009	Kinkajou	Educationalorganisation	1 case	Adult	Bitten onfinger	Dom	Case was bitten by a wild-born petkinkajou (a rainforest mammal relatedto a raccoon) from an educational organisation.The animal died shortly afterwards.Blastomycosis DNA sequences from the patient isolate andkinkajou tissues were indistinguishable.	[31]
Cowpox	France	2011	Rats	Pet store	1 case	Adult	Directcontact	Dom	Case fell ill after buying two ratsfrom a pet store. Other rats from thestore had died but were not investigated.	[32]
Cowpox	Germany	2009	Rats	Pet shop	5 cases	2 × child, 3× adult	Directcontact	Dom	Five cases occurred in two familiesthat had purchased rats from thesame pet shop. Some of the ratsdeveloped skin lesions after purchase.	[33]
Cowpox	France	2009	Rats	Pet store; pet breeder	4 cases	1× child, 3× adult	Scratches	Dom	Four cases of infection from sick petrats from the same pet store. The humancases were shown to be infected by aunique cowpox virus strain. All fourpet rats died.	[34]
Cowpox	Germany	2008	Rats	Pet shops;wholesaler	6 cases	2× child, 4× adult	3× directcontact, 3× notspecified	Dom	Five cases of cowpox, and oneputative case, among pet rat owners.All had contact with rats recentlypurchased from pet shops thathad sourced from same wholesaler.	[35]
Cryptosporidiosis	USA	2007	Unknown	Pet shop	1 case	Adult	Directcontact	>1 category	A pet shop employee was infectedwith *Cryptosporidium* horse genotype.Case reported no contact withhorses although did have contactwith numerous other animals.	[36]
*Edwardsiella tarda*	USA	1981	Turtle	Pet shop	1 case	Adult	Oral	Dom	The patient was infected with*Edwardsiella tarda*, an organism associatedwith cold blooded animals. Patient’s son had recentlypurchased a turtle from a pet shop.Patient drank from a glass containingtank water. No specimens were available from turtle or tank.	[37]
Lymphocytic choriomeningitis virus (LCMV)	Romania	2008	Unknown	Pet shop	2 cases	Adults	Notspecified	Occ	A case of LCMV infection in a petstore worker, and evidence of a previous infection in oneother employee. No samples were takenfrom rodents at the store.	[38]
LCMV	USA	2005	Hamsters	Pet store; petdistributor	1 case(plus 4 secondary cases via a common organ donor)	Not specified	Notspecified	Dom	Organ donor exposed to LCMV by hamsterrecently purchased from a pet store(although there was no evidence of LCMVinfection in the donor). Illnessoccurred in four organ transplant recipients,3 of whom died. More LCMV-infectedhamsters were found in both the pet store and the distributioncentre. Phylogenetic analysis linked thehuman and animal infections, includingthe donor hamster.	[39]
LCMV	USA	1974	Hamster	Petdistributor	181 cases	Not specified: ages ranged from 2 to 74 years	Notspecified	Dom	181 symptomatic laboratory confirmed cases in persons withhamsters sourced from a single distributor. Breeder was anemployee of a biological products firm that hadpreviously been associated withoutbreaks of LCMV from hamsters used for tumor research.	[40]
LCMV	USA	1974	Hamster	Pet shop	6 cases	2× child, 4× adult	All directcontact, incl 2×bite	Dom	Two individuals living in same householdcontracted severe infection from ahamster (proven to have LCMV)ecently purchased from a local pet shop.Three additional members of thefamily and a neighbor had a mild illnesswith raised antibody titres to LCMV (all handled thehamster and its bedding).	[41]
Leptospirosis	UK	2006	Rats	Pet shop	1 case	Adult	Notspecified	Dom	Case purchased two pet rats from a petshop three months prior to falling ill.Leptospiral DNA was detected in both rats,and other rats from same litter.	[42]
Leptospirosis	Austria	2001	Unknown	Pet shop	1 case	Adult	Notspecified	Occ	Case worked in a petshop. No discussionof possible exposures.	[43]
Leptospirosis	USA	1971	Mice	Pet shop	1 case	Adult	Oral	Dom	Case of leptospirosis acquired from petmice recently purchased from a petshop. Infection may have been acquired when the case’sdaughter used his toothbrush to cleanthe mouse-cage.	[44]
Monkeypox	USA	2003	Prairie dogs	Pet store;distributor	20 cases (part of an outbreak involving 72 cases )	i) 11 cases: 3–43y, ii) 9 cases: 5× child, 4× adult	i) 11 cases: Alldirect contact, incl 2×scratch/bite, 3× openwounds, ii) 9 cases:not specified	i) 11 cases: >1 category, ii) 9 cases: >1 category	Outbreak of monkeypox,including two pet store employees and twoanimal distributors. Acquired from prairiedogs which entered the communitythrough pet shops and pet swap meets.Papers detail two clusters within theoutbreak: i) 11 cases and ii) nine cases.	[45], [46]
MRSA	Canada	2006	Cats	Rescuecentre	4 cases	Not specified	1× directcontact, 3× notspecified	>1 category	Two kittens from a rescue centre wereinfected with *Staphyloccocus aureus*. Some of theirlittermates had previously died of anunknown disease. Indistinguishablestrains were isolated from both owners,one veterinary employee (out of 24 people tested)and the operator of the rescue centre, as wellas another cat in the household.	[47]
Psittacosis	Brazil	2012	Unknown	Pet shop	1 case	Adult	Notspecified	Occ	Case contracted *Chlamydophila psittaci* after startingwork at a pet shop.	[48]
Psittacosis	Japan	2004	Birds	Pet shop	2 cases	Adults	Notspecified	Occ	An elderly couple who ran a pet shop(selling psittacine birds) contractedpsittacosis. No bird sampling wasconducted.	[49]
Psittacosis	Belgium	1988–2003	Birds	Breedingfacilities	7 cases	Adults	Notspecified	>1 category	*C. psittaci* DNA detected in 6/46owners of pet birds obtained from sixdifferent breeding facilities. All of thesehad birds that tested positive for *C. psittaci* by PCR or culture.A veterinary student working at thefacilities was also culture positive andhad mild illness.	[50]
Psittacosis	Japan	2001	Birds	Pet shop	2 cases	Adults	Notspecified	Occ	Cases worked in a pet shop where someparakeets had recently died. [Article in Japanese]	[51]
Psittacosis	Slovenia	Unclear: 1991–2001	Birds	Pet shops;breeders	9 cases	Not specified	Notspecified	Occ	Nine pet shop keepers/breeders(out of 86 pet shop keepers/breeders[10.5%]) were seropositive for*C. psittaci*. Second study from 1997of pet store salesmen, breeders, veterinaryemployees and employees in theanimal slaughter industry showedhighest seropositivity (18.2%) was found insalesmen from pet stores.	[52]
Psittacosis	USA	1980s	Birds	Pet shops	Unknown	Not specified	Notspecified	Occ	10% of psittacosis cases reported toCDC during the 1980s (where the source ofinfection was known) occurred in pet shopemployees.	[53]
Psittacosis	USA	1997	Birds	Pet stores	i) 1 case, ii) Unknown	Not specified	Notspecified	i) Dom, ii) >1 category	i) One individual with a positive antibodytitre was found amongst a groupof pet bird owners who were tested after the bird lot from which their pets came wasconfirmed to have chlamydiosis,ii) Birds from pet stores were tested for *C. psittaci* following illness in pet storeemployees and bird owners. Persons with high antibody levelshad been exposed to PCR positive birds.	[54]
Psittacosis	USA	1997	Unknown	Pet shop	1 case (also 7 secondary nosocomial cases)	Not specified	Notspecified	Occ	A pet shop worker was hospitalisedwith psittacosis.	[55]
Psittacosis	USA	1997	Bird	Petdistributor	1 case	Adult	Directcontact	Occ	A dealer in exotic animals became illafter handling a dead cockatiel.	[56]
Psittacosis	USA	1995	Birds	Pet stores;distributor	Unknown (35 households)	Not specified	Notspecified	Dom	Avian chlamydiosis detected in ashipment of >700 pet birds to a particulardistributor. Among people who purchasedbirds sourced from this distributor,evidence of transmission of psittacosis was foundin 35 (30.7%) households when clinical andserological case definitions were combined.	[57]
Psittacosis	Spain	1993	Birds	Pet shop	4 cases	Not specified	2× directcontact, 2× notspecified	Dom	Two cases each bought a parakeet at thesame pet shop. Additional serological evidence of infectionin two of the cases’ relatives. [Article inSpanish]	[58]
Psittacosis	UK	1991	Birds	Pet shop	7 cases	1× child, 6× adult	Notspecified	>1 category	An outbreak of seven cases of *C. psittaci* originating from a local pet shop. Allcases had links to the shop, and three wereemployees. The shop had recently takendelivery of four love-birds, two of whichhad been unwell and died. None of thebirds were tested.	[59]
Psittacosis	Sweden	1977	Unknown	Pet shop	1 case (also 11 secondary cases, of which 9 nosocomial)	Adult	Notspecified	Visit	Case visited two pet shops prior to his(fatal) illness. Two parrots in the shopshad been bought from a wholesalerconnected with a previous outbreak[60], but attempts to isolate chlamydiaefailed. Eleven secondary casesoccurred.	[61]
Psittacosis	Japan	1976	Birds	Pet shop	1 case	Adult	Notspecified	Visit	Case visited a pet shop 11 days prior tofalling ill with psittacosis.[Article in Japanese]	[62]
Psittacosis	UK	1974	Birds	Pet shop	3 cases	1× adult, 2× not specified	2× directcontact, 1× notspecified	Occ	The owner of a pet shop became illafter acquiring parrots from a dealerconnected with a previous outbreak[63]. A second shipment of parrots was kept in thesame cage. One parrot died; two peoplewho had cared for it fell ill withcompatible symptoms.	[64]
Psittacosis	UK	1973	Birds	Privatepet distributor	3 cases	Not specified	Notspecified	>1 category	A pet distributor and a husband-wifecouple fell ill after being in proximityto a sick parrot.	[63]
Psittacosis	Sweden	1967–1969	Birds	Pet shop	18 cases	Not specified	Notspecified	>1 category	13/24 cases of ornithosis were probably infected from the same petshop and five more got their birds from awholesale dealer who provided birds to thepet shop. Attempts to culture from thebirds were not successful.	[60]
Psittacosis	Sweden	1963	Birds	Pet shop	13 cases	1× child, 12× adult	Notspecified	>1 category	13 cases of ornithosis were associated with a pet shop. Birds at the shop were culture positive for *C. psittaci*.	[65]
Rat bite fever	USA	2004	Rat	Pet shop	1 case	Adult	Finger woundfrom cage	Occ	Pet shop employee sustained a minorfinger wound from a rat cage and diedfrom sepsis and multi-organ failure 59days later.	[16]
Rat bite fever	UK	2001	Rat	Pet shop	1 case	Child	Bitten onfinger	Visit	A case of septic arthritis of the hip in a teenager following a bite on the finger from a rat in a pet shop. *Streptobacillus moniliformis* was cultured from joint fluid.	[17]
Ringworm	Japan	2006	Unknown	Pet shop	1 case	Adult	Directcontact	Occ	A case of tinea corporis (*Arthroderma benhamiae*) in a petstore employee. Likely that patient wasinfected through contact with ananimal in the pet shop where she handledsmall animals.	[11]
Ringworm	Japan	2002	Unknown	Pet shop	1 case	Not specified	Not specified	Occ	Pet shop worker with *Arthroderma* *benhamiae* lesions on face and hand,unknown exposure. [Article in Japanese.]	[12]
Ringworm	Japan	2002	Hedgehog	Pet shop	1 case	Adult	Notspecified	Dom	Case had a lesion on her palm. Hadbought a hedgehog from a pet shop fouryears prior. Isolates from the patient andhedgehog were identified as*Trichophyton mentagrophyes* var. *erinacei*.	[13]
Ringworm	Slovakia	2002	Guinea pig	Zoo	2 cases	1× adult, 1× child	Notspecified	Dom	Two cases of infection in a family whichkept a guinea pig obtained from a zoo.Samples from cases and guinea pig wereidentified as *T. mentagrophyes* var. *quinckeanum*.	[66]
Ringworm	USA	2000	Hedgehogs	Pet store	3 cases	Adults	1× directcontact, 2×not specified	>1 category	Three patients developed culture positive ringwormafter handling or purchasing African pygmy hedgehogsfrom pet stores. Two isolates were atypical*Trichophyton mentagrophytes* andone was *T. mentagrophytes* var *erinacei*.	[67]
Ringworm	Japan	1991	Dog	Pet shop	1 case	Adult	Notspecified	Dom	Case purchased a puppy from a petshop four weeks before presenting withsymptoms. The puppy was asymptomatic,but *Microsporum canis* was isolated from both case and puppy.	[14]
Salmonellosis	USA	2009–2011	African dwarf frogs	Breeder;petdistributor	56 cases	Not specified	Notspecified	>1 category	56/86 patients with *Salmonella* Typhimurium whowere interviewed had recent contact with African dwarf frogssourced through two distributors from thesame breeder. These cases were amongst224 reported with a unique strain.	[68]
Salmonellosis	USA	2007	Turtles	Pet store	16 cases	Not specified (for the 16 linked to pet stores)	Not specified (forthe 16 linked to petstores)	Dom (possibly with additional exposures)	16/78 cases with *S.* Java who wereinterviewed had recent exposure to turtlespurchased in retail pet stores. Samplescollected from six turtles (or theirhabitats) yielded the outbreak strain.These cases were amongst 107 infected with thesame strain of *S.* Java.	[69]
Salmonellosis	USA	2004	Rodents	Petdistributors	13 cases	Not specified	Not specified	Dom	13/22 cases of *S.* Typhimurium who were interviewed had exposure to rodentspurchased from pet stores. Sevendistributors were identified but nosingle source was found. These caseswere amongst 28 reported withmatching isolates.	[70]
Salmonellosis	Canada	2000–2003	Fish	Pet shops	33 cases	Not specified	Notspecified	Dom	*S.* Java was detected in 8/34 pet shops fromwhich 33 individuals with *S.* Java infectionhad purchased tropical fish.	[71]
Salmonellosis	USA	1999–2000	Cats	Rescueshelter	4 cases (and two secondary cases)	Not specified	Notspecified	Dom	Four people with *S.* Typhimurium infection adoptedkittens from an animal shelter. Isolates fromnine adopted cats from the shelter wereindistinguishable from the human isolates by PFGE. Two secondary cases occurred. (Onefurther human isolate was found to have thesame PFGE pattern, but no connection tothe shelter.)	[72]
Salmonellosis	Ireland	1999	Terrapins	Pet shop	8 cases	7× child, 1× adult	Not specified	Not specified (either dom or “close contact”)	Eight cases of *S.* Tel-el-kebir had contactwith pet terrapins purchased from thesame pet shop.	[73]
Salmonellosis	Canada	1995–1997	Pygmy hedgehogs; sugar gliders	Stockfarm; breeders	10 cases	9× child, 1× adult	1× directcontact, 9× notspecified	>1 category	Nine cases of *S.* Tilene had contact with families owningAfrican Pygmy hedgehogs, and one case’s family ownedsugar gliders. The sugar gliders and all but one of thehedgehogs had been directly acquired frombreeding herds or stock farms. In most cases,*S.* Tilene was isolated from the implicatedanimals or animals from the same breeders.	[74], [75]
Salmonellosis	USA	1994	Iguana	Pet stores; pet show	Unknown(17 households)	Not specified	Notspecified	Dom	25/32 *S.* Marina cases had a history ofexposure to an iguana in the week beforeillness. Of these, cases from sixteenhouseholds obtained their iguana from a pet store and oneobtained theirs from a pet show.	[76]
Salmonellosis	USA	1994	Hedgehogs	Breeders	1 case	Child	No directcontact	>1 category	A case of *S.* Tilene in a 10-month old babywhose family owned a breeding herd of 80African Pygmy hedgehogs. One ofthree hedgehogs tested yielded *S.* Tilene.	[77]
Salmonellosis	Japan	1985	Turtle	Pet shops	2 cases	1× adult, 1× child	Notspecified	Dom	Two cases of *S.* Paratyphi B occurredin a family who had a pet turtle positive for the same organism.Investigations also detected this pathogen in turtles or turtletanks in 4/12 pet shops in the city.	[78] **
Salmonellosis	USA	1983	Turtles	Pet shops	12 cases	11× child, 1× adult	1× directcontact, 11× not specified	Dom	12/83 cases of *Salmonella* had ahistory of exposure or probable exposure toturtles from petshops. Turtles were collected from pet shops inPuerto Rico and pooled into ‘lots’ for testing; all lotsincluded at least one animal that wasculture-positive for *Salmonella*.Contamination is believed to haveoccurred at the turtle farm prior to distribution.	[79]
Salmonellosis	USA	1970–1971	Turtles	Pet shops;department store	i) 2 cases, ii) 36 cases (possibly more, but not stated)	i) 2× child, ii) not specified	Notspecified	Dom	i) Case study of two siblings with *S.*Hartford infection from a pet turtle(also positive for *S.* Hartford) purchased at a department store,ii) Also report of six surveys of laboratory-confirmed cases ofsalmonellosis, where 193/1239 patients with salmonellosis ownedpet turtles (it was noted that all theturtles from one survey (36 patients)came from pet shops or department stores).	[80]
Toxocariosis	USA	1989	Dog	Pet store	1 case	Child	Notspecified	Dom	Young girl suffered permanent loss ofvision due to ocular toxocariasis after herparents purchased a puppy from a petstore.	[81]
Tularemia	USA	2002	Prairie dogs	Petdistributor	1 case	Adult	Directcontact	Occ	61 prairie dogs at a pet distributor testedpositive for *Francisella tularensis*. An animalhandler at the facility showed serologicalevidence of recent infection.	[82]

*Occ = occupational (exposure associated with case’s place of work); dom = domestic (pet owned by case or relative/friend of case), visitor = case visited place of likely exposure, outside of domestic setting).

**The original source paper for this incident (Murao T *et al* (1985) Ann Rep Fukuoka City Inst Hyg Environ, 10, 70–71) is only available in Japanese. The paper by Nagano contains sufficient information to include the incident in this review.

Table 1 therefore summarises the cases of disease associated with a pet shop that were identified by the literature review. Fifty seven cases of disease or incidents associated with pet shops or other facilities distributing companion animals were included. Bacterial, viral and fungal diseases were all identified, and ranged in severity from mild to life threatening. For example, infection with ringworm (Dermatophytosis) was noted in several articles, with four separate examples in Japanese pet shop employees and customers [11]–[14]. Zoophilic dermatophyte infections are rarely serious, generally self-limiting and respond well to treatment [15]. In contrast, two articles describing infection with rat bite fever (*Streptobacillus moniliformis* or *Spirillum minus*) were identified by the review [16], [17], one of which occurred in a pet shop employee and resulted in his death. Rat bite fever has a mortality rate of up to 13% in untreated cases [18].

The infection described most often was psittacosis (n = 18), followed by salmonellosis (n = 12) (Table 2). All of the psittacosis infections were associated with birds (where the putative animal source was identified), and no other avian infection was recorded in the review. The next group of animals most commonly referenced were rodents (n = 11), including rats, mice and prairie dogs. Four papers reported that the infections occurred through scratches or bites, two through oral transmission, one through a wound from a rat cage, and seven through other direct contact (including one paper with cases infected by a mixture of bites and direct contact). The review also included one paper (detailing a salmonellosis infection) which specified that the case had had no direct contact with the pet. In the remaining papers the method of transmission was not specified for some or all of the cases (n = 42). This includes 17 of the 18 papers reporting psittacosis incidents; it is likely that many of these infections occurred via airborne transmission.

**Table 2:** Incidents/outbreaks identified by the review, by zoonotic agent and animal category.

Zoonosis/agent	Birds	Cats/dogs	Hamsters/guinea pigs	Hedgehogs	Rodents	Turtles	Other	Not known	Total
LCMV	0	0	3	0	0	0	0	1	4
Leptospirosis	0	0	0	0	2	0	0	1	3
Pox virus	0	0	0	0	5	0	0	0	5
Psittacosis	15	0	0	0	0	0	0	3	18
Ringworm	0	1	1	2	0	0	0	2	6
Salmonellosis	0	1	0	1	1	4	5	0	12
Other	0	3	0	0	3	1	1	1	9
Total	15	5	4	3	11	5	6	8	57

Thirty of the papers referenced incidents in the Americas, nineteen referenced incidents in Europe, and eight referenced incidents in South East Asia. The majority of the papers described individual case reports or outbreaks of fewer than ten cases associated with pet shops (or other locations selling/distributing companion animals) (n = 42), with only three describing outbreaks with 50 cases or more (an outbreak of lymphocytic choriomeningitis virus in hamsters, an outbreak of monkeypox in prairie dogs, and an outbreak of salmonellosis in African dwarf frogs). Twenty-two of the incidents involved adults only, three involved children only, 11 involved both adults and children, and 21 did not specify the age of some or all of the cases.

Thirty-five papers described an incident associated with a pet shop, eight were associated with a breeder or distributor, five with some other facility (an animal shelter, an educational organization, two rescue centres, and a zoo; all of which sold or distributed animals to members of the public), and the remaining nine incidents involved more than one type of facility (most commonly involving both a distributor and pet shop). Twenty-five of the papers involved infections occurring in a domestic setting, fourteen in an occupational setting and three described infections occurring after a visit to a pet shop. Fifteen papers covered outbreaks where the cases fell into more than one category or where the setting was unspecified.

## Discussion

Pet shops can play an important role in the control of zoonotic infections from companion animals. They are the initial point at which members of the public can access information and advice on the risks associated with their newly purchased pets. Unfortunately, there is evidence to suggest that pet shop employees do not adequately understand or control the risks. A 2003 poll (commissioned by The Royal Society for the Prevention of Cruelty to Animals) of 300 pet shops which reported trading in exotic pets, asked pet shops whether any illnesses contracted by a client’s prospective pet could be passed onto humans; 36% answered “No, not at all” [19]. It is important that zoonotic risks are recognized and addressed because the consequences of these infections can be very serious.

The systematic literature review described in this manuscript identified 82 papers covering 57 separate human infections, outbreaks or incidents believed to have been associated with pet shops. Although the review was conducted in a systematic manner, the authors acknowledge that this list is not comprehensive; in order to be comprehensive, individual searches would have to be conducted for each potential zoonotic disease, and zoonotic incidents are often not written up in peer-reviewed journals. However, the review does present a representative sample of papers derived from a well-defined set of search criteria.

A wide spectrum of infections acquired from pet shops was identified by the review. Salmonellosis and psittacosis were the most commonly documented diseases, however more unusual infections such as tularemia were also identified. Many of the references relate to infections in pet shop employees, where often the precise source of infection was undetermined but the pet shop was assumed to be involved. The animals involved in the transmission of these infections were varied, including birds, mammals and rodents, and cover both common household pets, such as dogs and cats, and more exotic creatures, such as iguanas and prairie dogs. Some zoonotic infections were associated with a variety of different companion animals (e.g. salmonellosis), whereas others were associated with only a narrow range of species (e.g. psittacosis). Whilst some of the pathogens identified in Table 1 are commonly foodborne (e.g. *Salmonella*), or transmitted by other established routes of zoonotic infection, e.g. bites and scratches, this review demonstrates that more unexpected routes exist, and that transmission through animal contact should be considered when defining strategies to prevent disease in the population.

There are other organisms which have been identified in pet shop animals, and which have the potential to cause human infection, but which were not identified in this literature review. For example, infections caused by *Yersinia pseudotuberculosis* and *Y. enterocolitica* may be contracted from pet rodents, however this is uncommon because the usual serotypes found in rodents do not affect humans. The lack of clinical signs in animals affected by these infections may increase the likelihood of transmission of the organism from pet to human; guinea pigs are commonly infected with *Y. pseudotuberculosis* and clinical signs are usually subacute, similarly *Y. enterocolitica* is usually asymptomatic in rodents [20]. It is also likely that other zoonotic organisms may have passed from pet shop animals to humans and caused disease, but have not been documented because of under-diagnosis and under-reporting, and a lack of follow-up of sporadic infections, e.g. cryptosporidium, giardia.

There are some diseases which were unexpected omissions in this review, e.g. pasteurellosis. A number of articles concerning pasteurella infections were initially accepted into the review on the basis of their abstracts, however they were not included in the final results because they did not specifically refer to pet shops. This might reflect a publication bias; because infections with *Pasteurella* spp. are commonly associated with animal exposures, case studies might not be written up in the literature. In addition, the association of pasteurellosis with cat and dog bites is very well established, so where articles on pasteurella infections do occur, links to pets and pet shops may not be deemed to be of sufficient interest to warrant inclusion in the final publication. Similarly, this may explain why the literature review included only one article on cat scratch disease. It is therefore important to note that unusual and unexpected zoonotic infections may be over-represented in peer-reviewed publications, and in this review.

A further limitation of this review was its restriction to English-language papers. Although a small number of foreign-language manuscripts were included where a translated abstract was available and provided sufficient information to fulfill the inclusion criteria, 137 out of 142 foreign-language papers were nonetheless excluded. The countries associated with incidents in this review (predominantly the Americas and Europe), reflect this bias. This may imply that pathogens that are more common in non-Western countries, or in more exotic animals not common in Europe and the Americas, were under-represented.

Incidents of rabies tracebacks and zoonoses from pet food were excluded from this review. They are nonetheless important public health considerations and can require a large amount of resource to deal with appropriately. For example, in the US in 1994, significant numbers of people were exposed to a rabid kitten in a pet shop and, although no human cases resulted, the final cost of the investigation and prophylaxis was estimated to be over $1 million with 665 people receiving prophylaxis [21], [22]. Such incidents are not necessarily unusual, and Rotz *et al.* summarise 22 large-scale incidents of exposure to rabid or presumed rabid animals (defined as administration of PEP to 25 or more people after an exposure) that occurred in the US between 1990 and 1996 [23]. The increase in *Salmonella* Typhimurium, designated definitive type 191a (DT191a), was an example of an outbreak from pet food detected in the UK in December 2008. The increase was found to be associated with raw frozen mice used as reptile feed and sold through wholesalers and distributors [24]. Revised infection control guidance for reptile owners and handlers has been published on the Health Protection Agency (HPA) website [25]. It is therefore important to note that there will be further significant events associated with pet shops beyond those summarized in this manuscript, which must be kept in mind when considering the importance of such facilities in the zoonotic transmission of disease.

While many zoonotic infections associated with pet shops are likely to result in single cases or familial incidents, e.g. rat bite fever, such premises also have the potential to amplify the risk of spread. A sick animal in a pet shop can potentially transmit the illness to other animals within the shop, and therefore to a large number of new pet owners, who may be geographically dispersed. Pet shops (and other locations that sell animals) can additionally act as a type of leisure activity, with families visiting to see and handle the animals, and potentially becoming exposed to zoonotic diseases even though they do not own a pet of their own. As such, pet shops can be the focus of very large outbreaks of disease, such as the 2003 incident in the USA where prairie dogs infected with monkeypox were widely disseminated through pet shops and pet swap meets, and resulted in over 50 cases of human disease. Such disease outbreaks can have a significant public health burden in the direct morbidity and mortality to cases, in financial and logistical impacts on laboratories and healthcare providers, and in the time and expertise required to investigate exposures and follow up potentially infected animals and human cases and contacts. The precise public health impacts will vary according to the zoonosis and the size of incident.

Given their potential to spread zoonotic infections, it is important that pet shops act to minimise the risk as far as possible. The current legislative framework is biased towards animal welfare in the UK, with few recommendations seeking explicitly to protect human health. However, those exposures that fall within occupational health and safety are an exception: employee safety is covered by health and safety at work legislation, and the Control Of Substances Hazardous to Health (COSHH) regulations additionally cover the health of other people who may be exposed to hazards in the workplace, including customers.[26]–[28] Local Authorities have powers to impose conditions on the licensing of pet shops, and most adopt model standards published by the Local Government Association which includes taking all reasonable precautions to prevent the outbreak and spread of disease [29]. Whilst proposing specific recommendations to improve control measures associated with companion animals in pet shops is beyond the scope of this paper, legislative authorities might consider more stringent oversight of pet breeders and distributors before animals enter the market. Alternatively, practical hygiene measures similar to those implemented on farms open to the public could be made mandatory in pet shops, and information leaflets on zoonotic risks and prevention measures for prospective pet owners could be provided to help to reduce the risk of infection.

## Supporting Information

Checklist S1 **PRISMA 2009 Checklist.**
(DOC)
